# A Randomized Study Comparing Ultrasound-Guided Internal Jugular Vein Cannulation Using Two Techniques: Short-Axis Out-of-Plane With Dynamic Needle Tip Positioning Versus Long-Axis In-Plane

**DOI:** 10.7759/cureus.63004

**Published:** 2024-06-23

**Authors:** Mainak Baidya, Mamta Sinha, Mayank Kumar, Nandkishore Agrawal, Sarita Ramchandani, Gade Sandeep, Swati Vijapurkar

**Affiliations:** 1 Anesthesiology, All India Institute of Medical Sciences, Raipur, Raipur, IND; 2 Anesthesiology, Critical Care, and Pain Medicine, All India Institute of Medical Sciences, Raipur, Raipur, IND; 3 Cardiac Anesthesia, All India Institute of Medical Sciences, Raipur, Raipur, IND

**Keywords:** first pass success, ijv cannulation, ultrasound-guided, dynamic needle tip positioning, short axis technique, long axis technique

## Abstract

Introduction

Internal jugular vein (IJV) cannulation is a routine procedure in operating rooms, critical care units, and perioperative settings. Ultrasound guidance has notably increased the success rates of IJV cannulation. A modified ultrasound technique known as the short-axis out-of-plane method with dynamic needle tip positioning (DNTP) allows for continuous visualization of the needle tip throughout the procedure. This study aims to compare the first-pass success rate of IJV cannulation using the DNTP and long-axis in-plane (LAIP) approaches.

Methods

One hundred patients between 18 and 70 years undergoing elective surgery requiring IJV cannulation were recruited. Patients were assigned randomly to the DNTP group (n = 50) or the LAIP group (n = 50). We recorded the first-pass success rate, time to achieve successful cannulation, number of skin punctures, overall success rate within five minutes, and potential complications such as pneumothorax and hematoma.

Results

The first pass success rate was higher in the DNTP group (48/50, 96%) as compared to the LAIP group (38/50, 76%, relative risk, 1.67; 95% confidence interval, 0.039-0.707; p = 0.008). The cannulation time was shorter in DNTP (116.98 ± 22.90 seconds) versus the LAIP group (213.04 ± 52.08 seconds; p < 0.001). No complications like pneumothorax or hematoma were noted in both groups.

Conclusion

We conclude that the ultrasound-guided DNTP technique for IJV cannulation, as compared with the LAIP technique, may significantly improve the first attempt cannulation, number of attempts, and cannulation time.

## Introduction

Internal jugular vein (IJV) cannulation is a commonly performed procedure in operation rooms, critical care units, and perioperative periods. It is often used for monitoring volume status, infusion of drugs, and inotropes. Traditionally, IJV cannulation was performed by landmark-guided technique but the introduction of an ultrasound (US)-guided technique has proven to accelerate the rate of success and to a decline in complications.

The American Society of Echocardiography and Cardiovascular Anesthesiologists has advocated the real-time use of US for central venous catheter (CVC) cannulation in the IJV [1]. Practice guidelines from the American Society of Anesthesiologists (ASA) have recommended static US imaging electively for identifying the anatomy before puncture and to locate the vessel and real-time US for puncturing the IJV [2]. An international expert panel recommends using two-dimensional US imaging for IJV cannulation when placing CVCs in ICU patients [3].

Various ultrasound-guided approaches exist for cannulating the IJV, including the long-axis in-plane (LAIP) and short-axis out-of-plane (SAOP) techniques. However, there is an ongoing debate over which method provides superior advantages [4]. The short-axis technique provides clear visualization of the artery and vein during cannulation, including the inter-relationship with adjacent structures. However, it may not offer continuous visualization of the needle tip. Dynamic needle tip positioning (DNTP) is an innovative ultrasound guidance technique, a variation of the conventional SAOP approach, enabling the needlepoint to be continuously visible [5]. Clemmesen et al. were the first to publish the DNTP approach for vascular access in phantoms [6]. Goh et al. successfully utilized the DNTP technique for cannulation, recommending its early adoption for challenging arterial cannulations [5]. In comparison to blind palpation or the LAIP technique, several prior studies have shown that the DNTP approach has a greater success rate for peripheral and central vascular cannulation [7-9].

The primary objective of this study was to evaluate and compare the first-pass success rate of IJV cannulation between DNTP using the SAOP technique and the LAIP technique. The secondary objective of this study was to know the number of skin punctures, time to guidewire insertion, cannulation time, overall success rate within five minutes, and any associated complications like pneumothorax or hematoma. 

This article was previously presented as a meeting abstract at the Annual Congress of the Association of Anaesthetists in Edinburgh, Scotland, on September 15, 2023.

## Materials and methods

After receiving approval from the Institute Ethics Committee (IEC) of AIIMS, Raipur, under proposal number AIIMSRPR/IEC/2021/744, this study was prospectively registered in the Clinical Trials Registry-India with registration number CTRI/2022/02/039907.

In this prospective, randomized clinical study, after taking written and informed consent, 100 patients of either sex, aged between 18 and 70 years posted for elective surgery requiring IJV cannulation were included. Patient refusal, patients with coagulopathy, presence of thrombus in IJV, infection over the insertion site, and trauma to the neck were excluded.

Sample size estimation

The sample size calculation was derived from a prior study [10], which documented first-pass success rates of 98% for the SAOP technique and 78% for the LAIP technique in IJV cannulation. Assuming a similar difference between the study groups, a power analysis with 80% power and a type I error of 0.05 indicated that 46 participants were needed in each group. To account for an estimated 10% dropout rate, the sample size was increased to 50 per group, resulting in a total of 100 participants.

A computer-generated random number table with a block size of 4 was used to randomly divide the patients into two groups of 50. The random sequences were inserted into opaque, sealed envelopes with sequential numbers.

An intravenous line was established in the operating room and ASA standard monitors were attached. General anesthesia was then induced with intravenous fentanyl (2 μg/kg) and propofol (2-2.5 mg/kg) and neuromuscular blockade was achieved using vecuronium (0.8-1 mg/kg). Following intubation, the patient was positioned in the Trendelenburg (20-30 degrees) position with the head slightly rotated to the contralateral side for cannulation. The cannulation technique was determined by opening the opaque randomization envelope. All cannulations were performed using 7 French triple-lumen catheters via the Seldinger technique in the right IJV by a single anesthesiologist with experience of a minimum of 100 prior ultrasound-guided cannulations.

**Figure 1:**
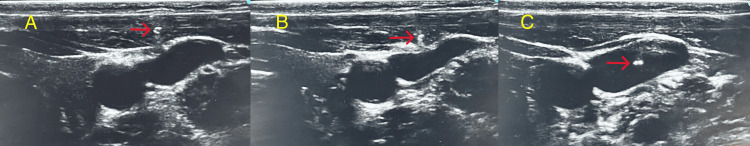
IJV cannulation using the DNTP technique The figure shows the advancement of the cannula in IJV. A: The hyperechoic needle tip (arrow) is under the skin near the IJV, B: The needle tip is at the wall of the IJV, C: The needle tip is advanced to the center of the IJV. DNTP: dynamic needle-tip positioning

For the DNTP technique, a SAOP view of the IJV was obtained at the level of the cricoid, and then at a 45-60-degree angle, the needle was advanced through the skin until the US scan showed the hyperechoic needle tip. After that, the probe was slid away from the needle insertion site until the needle tip was not visible. Again, the needle was moved forward a few millimeters until the US image showed the needle tip again. This stepwise process was repeated many times until the needle tip was visualized in the IJV (Figure 1).

After confirming the backflow of venous blood in the syringe, the guidewire was placed through the needle into the vein. Further, the triple-lumen catheter was inserted inside.

For the LAIP technique, a transverse SAOP image of the IJV was acquired with the US probe positioned at the level of the cricoid. The probe was rotated by 90 degrees to achieve a longitudinal view of the IJV, ensuring that the IJV remained centered within the ultrasound image at all times. The needle was inserted at an angle ranging from 30 to 45 degrees, maintaining parallel alignment with the ultrasound probe. Confirmation of the needle's entry into the IJV was established by observing the backflow of blood into the needle. After the guidewire was threaded through the needle into the vein, the triple-lumen catheter was advanced into position.

The first pass success rate of IJV catheterization was defined as the successful placement of a CVC with a single needle pass through the skin, without needing to redirect the needle. The time to guidewire insertion was the time from the beginning of skin penetration till the guidewire was ultrasonically confirmed to be visible in the IJV. Cannulation time was defined as the time from the beginning of skin penetration to blood aspiration from the CVC port. If the operator could not insert a CVC within 5 minutes after the initial skin puncture, the attempt was considered a failed cannulation.

Lung ultrasonography (USG) was done just after CVC insertion and in the postoperative area to rule out pneumothorax. The tip position of the CVC was verified by performing a chest radiograph. Any other complication like the presence of hematoma was noted.

Statistical analysis

Data were recorded in Microsoft Excel (Microsoft Corporation, Redmond, WA, US) and analyzed using SPSS version 23.0 (IBM Corp., Armonk, NY, US). Continuous variables were presented as mean ± standard deviation or median (25th and 75th percentiles) while categorical variables were expressed as counts (percentages). The Kolmogorov-Smirnov test was used to assess normality. Ordinal variables and non-normally distributed continuous variables were compared between groups using the Wilcoxon Mann-Whitney U test, whereas normally distributed continuous variables were compared using the unpaired student's t-test. The relationship between categorical variables was examined using Fisher's exact test or the chi-square test. A p-value of less than 0.05 was considered statistically significant.

## Results

**Figure 2:**
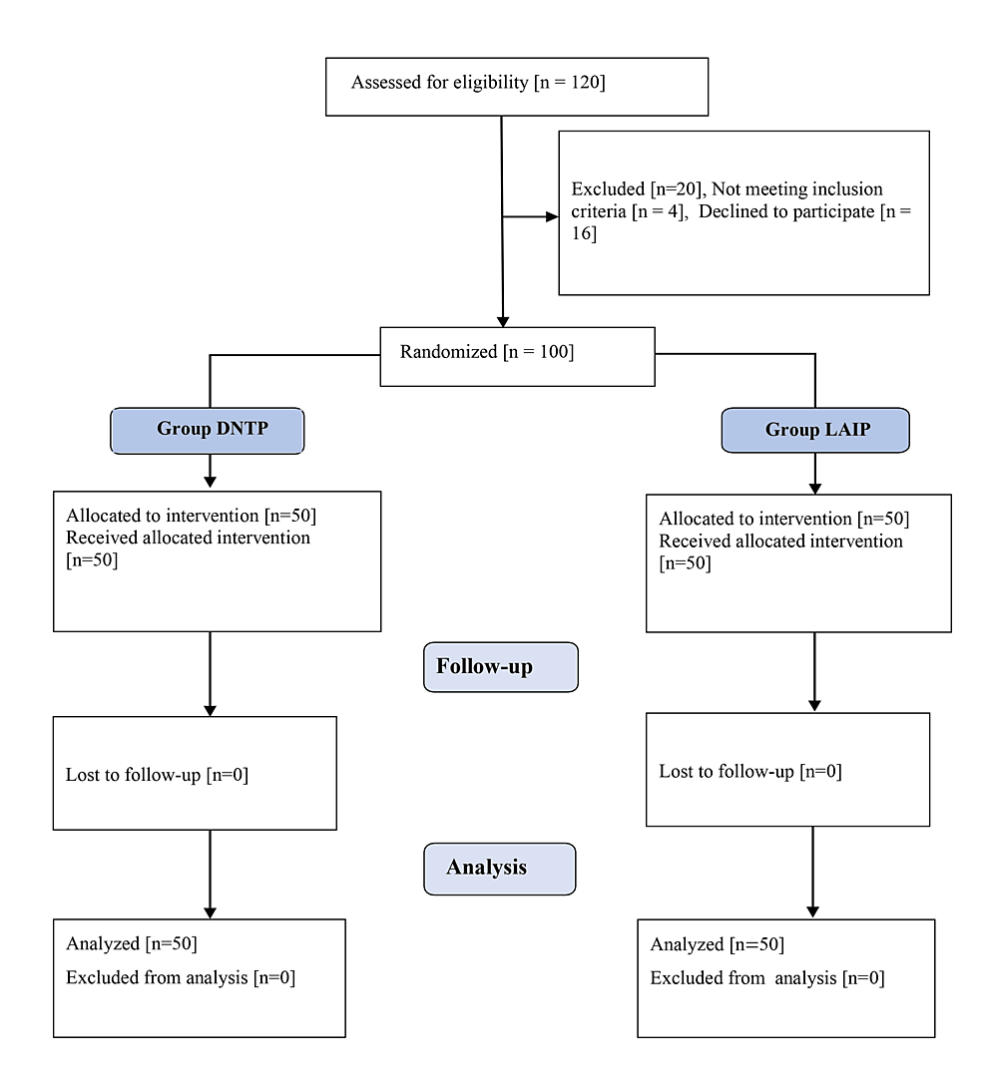
CONSORT diagram showing patient allocation, randomization, and analysis DNTP: dynamic needle-tip positioning, LAIP: long-axis in-plane, CONSORT: Consolidated Standards of Reporting Trails

A total of 100 participants (66 males and 34 females) were included in the study (Figure 2).

Demographic parameters like age, gender, height, weight, neck circumference, and body mass index (BMI) were comparable in both groups (Table 1).

**Table 1:** Demographic data of patients Data are presented as mean ± SD and n (%) Significant at p<0.05, *: t-test, #: chi-squared test, $: Wilcoxon-Mann-Whitney U test, BMI: body mass index, DNTP: dynamic needle-tip positioning, LAIP: long-axis in-plane

Parameters	Group		P value
	DNTP (n = 50)	LAIP (n = 50)	
Age (Years)	48.68 ± 9.48	48.48 ± 7.94	0.909^*^
Gender			0.398^#^
Male	31 (62.0%)	35 (70.0%)	
Female	19 (38.0%)	15 (30.0%)	
Height (cm)	164.98 ± 5.54	163.56 ± 7.56	0.287^*^
Weight (Kg)	65.16 ± 5.94	67.58 ± 6.34	0.052^*^
Neck circumference (cm)	35.58 ± 1.43	35.96 ± 1.31	0.177^$^
BMI (Kg/m^2^)	24.88 ± 1.30	25.16 ± 1.45	0.312^*^

The DNTP group demonstrated a significantly higher first-pass success rate compared to the LAIP group (96% vs 76%, relative risk, 1.67; 95% confidence interval, 0.039-0.707, p = 0.008), which was the primary outcome measure of this study (Table 2).

**Table 2:** First-pass success, number of skin punctures, and time to achieve successful cannulation (seconds) Data are presented as mean ± SD and n (%) Significant at p < 0.05, *: Fisher's exact test, #: Wilcoxon-Mann-Whitney U test, $: chi-square test, DNTP: dynamic needle-tip positioning, LAIP: long-axis in-plane

Parameters	Group		P value
	DNTP (n = 50)	LAIP (n = 50)	
First-pass success (Yes)	48 (96.0%)	38 (76.0%)	0.008^*^
Number of skin punctures			0.002^*^
1 Puncture	48 (94.0%)	36 (72.0%)	
2 Punctures	2 (4.0%)	14 (28.0%)	
Time to insert guidewire (seconds)	65.04 ± 17.07	112.26 ± 33.23	<0.001^#^
Time to achieve successful cannulation (seconds)	116.98 ± 22.90	213.04 ± 52.08	<0.001^#^
Overall success within 5 minutes (Yes)	50 (100.0%)	49 (98.0%)	1.000^*^
Pneumothorax (Yes)	0 (0.0%)	0 (0.0%)	1.000^$^
Hematoma (Yes)	0 (0.0%)	0 (0.0%)	1.000^$^

The DNTP group had a statistically significant lower number of patients requiring more than one skin puncture compared to the LAIP group (p<0.001).

There was a statistically significant difference in the time taken to insert the guidewire between the DNTP group (65.04 ± 17.07) and the LAIP group (112.26 ± 33.23), with p < 0.001. Additionally, the time for cannulation was significantly less for the DNTP group (116.98 ± 22.90) as compared to the LAIP group (213.04 ± 52.08) with p < 0.001 (Table 2).

Complications like pneumothorax and hematoma were not noted in any of the groups. Postoperative chest X-ray (CXR) follow-up confirmed that the central venous catheter was correctly positioned in all cannulations.

## Discussion

This prospective, randomized clinical study compared the IJV cannulation between DNTP using the SAOP approach and the LAIP technique. IJV catheterization is widely regarded as valuable for ionotropic and drug infusions in hemodynamically unstable patients, and it is commonly performed in operating rooms and intensive care units. Because of its superficial course, it is usually preferred. It can be compressed easily in case of accidental puncture of the carotid artery.

There are mainly two approaches for needling using ultrasound: the LAIP approach and the SAOP approach, both of which have their advantages and disadvantages. Previous research on the success rates of the two techniques has produced conflicting results [10-12].

The traditional SAOP technique enables target vessels and surrounding structures to be seen at the same time, making it easier to identify the needle tip’s orientation [13]. However, it has a higher risk of posterior wall penetration compared to the LAIP technique due to difficulties in distinguishing the needle shaft from the tip in the fixed perpendicular image. In contrast, the LAIP technique allows simultaneous visualization of the needle shaft and tip, leading to lesser posterior wall injury [14,15].

In the LAIP technique, visualizing the needle entirely within the image can be challenging. The midline axis of the target vessel's longitudinal plane and the entire needle length must be aligned with the narrow ultrasonic beam [16]. When the longitudinal image plane isn’t precisely parallel to the vessel wall, advancing the needle tip can raise the risk of unintended damage to the vessel wall and nearby structures [17].

Recently, a modified ultrasound technique where the tip of the needle is advanced in a stepwise fashion, i.e. DNTP using the short-axis approach has been described, which has been shown to be a more successful method of central venous catheterization. In studies conducted by Goh et al. [5] and Clemmenson et al. [6], it was reported that this technique allows precise visualization and localization of the needle tip by alternate advancement of the needle and the ultrasound probe using the SAOP approach. This method was found to be useful in cannulating peripheral vessels in both adult and pediatric populations.

The two stages of successful cannulation are puncturing the blood vessel, effectively introducing the guidewire without resistance, and inserting the cannula with backflow of venous blood through all three ports of the catheter. With the DNTP technique, the needle tip is consistently visualized under ultrasonic guidance and this could be the reason for the modified short-axis DNTP technique having a higher first-pass success rate as compared with the LAIP technique.

The principal objective of this study was to assess the first pass success rate of IJV cannulation by the DNTP approach and the LAIP approach. The first-pass success rate in the present study for the DNTP short-axis technique group was 96% as compared with the LAIP group (76%). The precise identification of the midpoint of the IJV on the DNTP view as compared with the LAIP view may have attributed to this clinical benefit. AlGhamdi et al. [18], in their study comparing short-axis versus long-axis ultrasound-guided techniques for IJV cannulation, reported the short-axis view to be advantageous over the long-axis view in terms of the first needle pass.

In our study, the time required for successful cannulation and median cannulation time was significantly less in the DNTP group (116.98 ± 22.90 seconds) as compared with the LAIP group (213.04 ± 52.08 seconds). This might be due to the less time required for localization of the midpoint of the IJV with the DNTP group as compared with the LAIP group.

In a study done by Chittoodan et al. [10], comparing the SAOP and LAIP techniques for IJV catheterization, including 99 models, the SAOP technique resulted in a significantly higher first-pass success rate (98%) as compared with the LAIP technique (78%). They took less time for cannulation in the SAOP technique as compared to the LAIP technique. Moreover, the number of needle passes was lesser in the SAOP group. They observed that the location of the carotid artery relative to the internal jugular vein was also difficult to assess in the long-axis view.

The results of our study are comparable to a similar study conducted by Seohee Lee et al. [7], which aimed to compare the first-attempt success rate of US-guided IJV cannulation between the DNTP and LAIP techniques. They found that the DNTP approach had a significantly higher first-pass success rate in cannulating IJV and needle redirection was significantly less than with the LAIP approach. This might be because the needle tip is under constant visualization in the modified short-axis technique (DNTP) compared with the long-axis in-plane technique (LAIP).

In a study by Rath A et al. [19], comparing the short-axis and long-axis approaches for IJV cannulation, it was found that cannulation is faster using the short-axis view. This is because focusing in the long-axis view takes longer and the target area is smaller.

Dynamic needle-tip positioning using the SAOP approach has also been compared with the LAIP approach for radial artery cannulation by Mesa BK et al. [8]. They found that the short-axis DNTP technique had a significant advantage as compared to the LAIP technique and that it resulted in higher first-attempt cannulation success and shorter cannulation time.

According to a study by P Patel et al. [20], the time required for guidewire insertion during IJV cannulation was shorter when using the short-axis approach (43.3 ± 5.64 seconds) compared to the long-axis approach (74.8 ± 39.36 seconds). Similar results were found in our study, with the guidewire insertion time being longer in the long axis than in the short axis approach.

Complications like hematoma or pneumothorax did not occur in our present study, as ultrasonic guidance facilitated the visualization and localization of the IJV due to experienced personnel performing the cannulations. Several studies on ultrasound-guided IJV cannulation have reported similar findings regarding complications [21,22].

Limitations

The anesthesiologist who performed cannulation was an expert in both imaging modalities. Consequently, the results may not apply to less experienced operators. Our study was conducted at a single center with 100 participants. Conducting a multicentric study with a larger sample size would likely produce more robust results. Our study did not include a comparison with the SAOP approach. This is a notable limitation, as including SAOP could have provided a more comprehensive assessment of the DNTP technique's effectiveness.

## Conclusions

We conclude that the ultrasound-guided dynamic needle-tip positioning (DNTP) technique for IJV cannulation, as compared with the long-axis in-plane (LAIP) technique, may significantly improve the first attempt cannulation success, reduce the number of attempts, and shorten the cannulation time. This improvement is likely due to better visualization of the needle tip with the modified short-axis out-of-plane (SAOP) DNTP technique compared to the LAIP technique. Complications such as hematoma and pneumothorax were not observed with either technique.
